# An evaluation of a low‐cost platelet‐rich plasma for osteoarthritis of the knee: A pilot study

**DOI:** 10.1002/jeo2.70420

**Published:** 2025-09-05

**Authors:** Daniel M. Cushman, Luke A. Johnson, Taylor Burnham, Richard Nelson, Jamie Egbert, Robert Burnham

**Affiliations:** ^1^ Department of Physical Medicine & Rehabilitation University of Utah Salt Lake City Utah USA; ^2^ Department of Orthopaedics University of Utah Salt Lake City Utah USA; ^3^ Calgary Alberta Canada; ^4^ Department of Internal Medicine University of Utah Salt Lake City Utah USA; ^5^ Central Alberta Pain and Rehabilitation Institute Lacombe Alberta Canada

**Keywords:** health economics, knee OA, low‐cost platelet‐rich plasma, osteoarthritis, PRP

## Abstract

**Purpose:**

To assess the characteristics and effectiveness of low‐cost platelet‐rich plasma (LC‐PRP) for knee osteoarthritis (OA) by evaluating its composition and effect on pain, function, satisfaction, safety and cost‐effectiveness.

**Methods:**

Level IV evidence single‐arm prospective cohort pilot study of 20 subjects (30 knees total) with mild‐to‐moderate knee OA. Two LC‐PRP injections were performed, 3 weeks apart. Platelet extraction/yield, patient‐reported outcomes and incidence of adverse events were assessed with a primary endpoint of 6 months. A cost‐effectiveness analysis of LC‐PRP compared to corticosteroid injection was conducted.

**Results:**

On average, this LC‐PRP preparation method allowed for recovery of 85% of platelets. Significant mean differences in Western Ontario and McMaster Universities Osteoarthritis Index (WOMAC) scores (total, pain and function) and global assessment score were observed at all follow‐up points compared to baseline (*p* < 0.05 for 1, 3, 6 and 12 months). Satisfaction was noted in 90% of patients at 6 months and 75% of patients at 12 months. No significant adverse events were reported. LC‐PRP was less costly ($654 vs. $1308) and yielded more QALYs (0.846 vs. 0.708) than corticosteroid injections. Per injection costs were estimated to be $10 for corticosteroid and $11 for LC‐PRP.

**Conclusions:**

This pilot study demonstrates that LC‐PRP ($11) may significantly benefit patients with knee OA. LC‐PRP appears to be a safe, cost‐effective means for improving pain and function in knee OA.

**Level of Evidence:**

Level IV.

**Abbreviations**
BMI: body mass index
CBC: complete blood count
CEA: cost‐effectiveness analysis
GPS: global patient satisfaction
Hb: haemoglobin
ICER: incremental cost‐effectiveness ratio
IRB: institutional review board
K‐L: Kellgren–Lawrence
LC‐PRP: low‐cost platelet‐rich plasma
OA: osteoarthritis
OARSI: Osteoarthritis Research Society International
PRP: platelet‐rich plasma
QALY: quality‐adjusted of life‐year
RBC: red blood cell
RCT: randomised controlled trial
SD: standard deviation
WBC: white blood cell
WOMAC: Western Ontario and McMaster Universities Osteoarthritis Index
WTP: willingness‐to‐pay threshold

## INTRODUCTION

Knee osteoarthritis (OA) is an extremely common and debilitating condition with an estimated global prevalence of 16% [13]. OA prevalence increases with age, with women impacted more commonly than men [13]. Among individuals ages 40 and older, the prevalence is 22.9%, compared to 49.8% among individuals ages 80 and older [13]. Knee OA affects 57.0% of women ages 80 and older compared to 38.9% of men ages 80 and older [13].

While current management of knee OA revolves around conservative treatments—biomechanical interventions, exercise, self‐management and education, oral or topical medications, strength training and weight management [34]—surgical management is considered the gold standard treatment in the form of a knee replacement. For injection therapies, corticosteroids remain the standard of care; approximately 84% of sports medicine physicians perform these knee injections at least monthly, reporting a median range of 11–20 injections per month [14]. Multiple detrimental effects of corticosteroid injections are well‐known, including alterations of the hypothalamic‐pituitary‐adrenal axis, blood glucose levels, bone turnover, inflammatory response, blood pressure and psychological well‐being [8, 20, 35, 51, 53]. Thus, effective alternative therapies are of utmost importance.

Platelet‐rich plasma (PRP) is an injectable preparation of a patient's blood, where the platelet portion of plasma is isolated and injected for a variety of pathologies. PRP has recently received significant attention for its potential application in treating pain and functional impairment due to knee OA. Systematic reviews of randomised controlled trials (RCTs) have demonstrated equivalent‐to‐superior treatment outcomes associated with the use of intra‐articular PRP compared to placebo, hyaluronic acid and corticosteroids [24, 38, 47]. Furthermore, the use of intra‐articular PRP is associated with a very low rate of adverse events [15] and is likely safer than injectable corticosteroids [20, 21, 22]. Notwithstanding its benefits, two major concerns have limited the wider use of PRP: (1) an uncertainty regarding the current evidence base due to study‐related bias, heterogeneity, and a lack of reporting standards [11, 25, 37, 49] (2) high cost [9, 33, 41].

As socioeconomic inequity directly relates to disability related to knee OA [2, 12, 28], limiting the cost of treatment is of utmost importance to ensure appropriate delivery of care to all patients. As PRP is currently not covered by the vast majority of public and private payors, patients are required to pay for the injections out of pocket, with an average cost of around $1000 per injection (as high as $2092) [9, 41]. Furthermore, research studies relating to PRP are often expensive due to the cost of the traditional method of formulating PRP, generally with commercial kits, and thus limited in scope.

The goal of this pilot study is to assess the characteristics and effectiveness of an easy‐to‐replicate low‐cost PRP (LC‐PRP) by evaluating PRP composition, effect on pain and function of OA knee injection, patient perception of change and satisfaction, safety via adverse event tracking, and cost‐effectiveness.

## METHODS

This study obtained institutional review board (IRB) approval (IRB_00173470) through the University of Utah IRB and was registered at ClinicalTrials.gov (NCT06184048). Between February and May 2023, consecutive patients eligible for a corticosteroid injection were enrolled from a single academic orthopaedic centre, and a prospective, single‐arm cohort pilot study was conducted. This study followed minimum reporting requirements for clinical studies evaluating PRP [11, 37].

### Sample

#### Sample size

Prior literature has demonstrated commercial neutrophil‐poor PRP kits produce extraction rates of around 0.30–0.60 (mean 0.45) [32]. Initial sample data on the LC‐PRP demonstrated a platelet extraction rate of 0.90. Thus, using a two‐sided significance of 0.05 and a power of 80%, only 20 subjects would be required to demonstrate superiority (Fischer's exact test) of the method over 0.45. Therefore, it was planned to treat 30 knees to account for attrition and patients who had bilateral knee OA. For clinical endpoints, prior systematic‐review evidence suggests that PRP provided functional improvement of 1.6x MCID [48]; thus a sample of 11 subjects would show improvement over control.

#### Inclusion criteria

All participants were adult patients with symptoms and clinical findings compatible with knee OA, Kellgren‐Lawrence (K–L) score 1–3 knee OA based on radiographs within the last 6 months, and who had failed at least 6 weeks of conventional conservative treatments (medication, physical therapy, physician‐directed exercise, etc.). Patients with bilateral knee OA were eligible to include both knees only if both knees fit the criteria.

#### Exclusion criteria

Knee surgery within the last 2 years, prior orthobiologic injection into the knee, major axial deviation greater than 30 degrees, thrombocytopenia or other haematologic disease, active systemic or local infection at the site of injection, nonambulatory patients, patients seeking care with active litigation pending, body mass index (BMI) over 40, and recent corticosteroid injection (6 months) or consumed oral steroids (3 months).

### Study design

#### LC‐PRP preparation method

**Figure 1:**
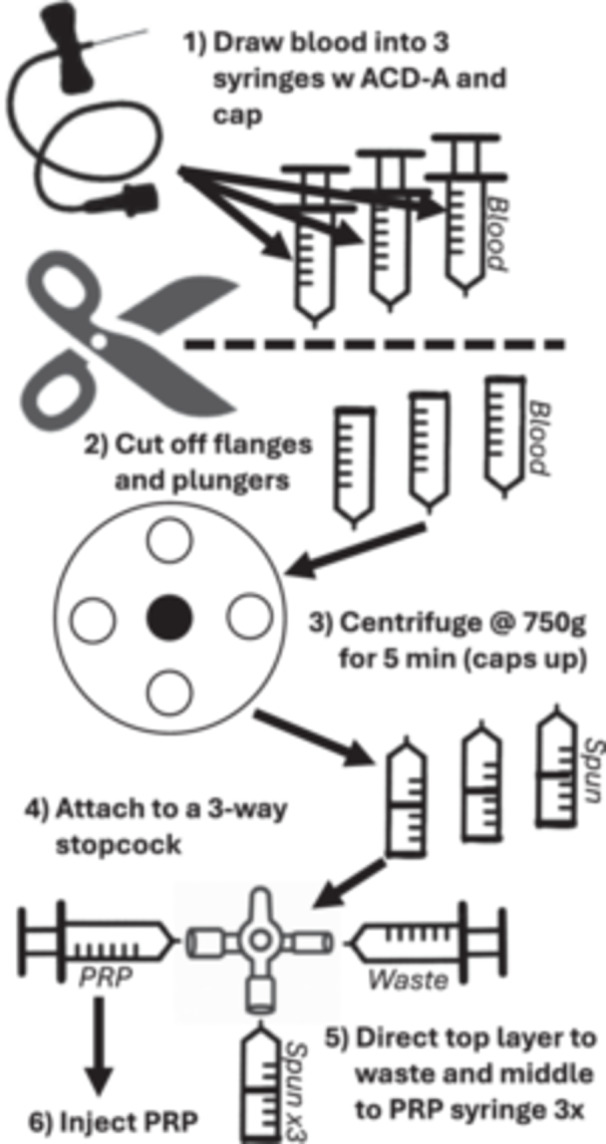
Schematic of low‐cost platelet‐rich plasma preparation technique. PRP, platelet‐rich plasma.

Please see Figure 1 (Supporting Information S1: Appendix 1 has the full methodology). Blood was never exposed to ambient air other than capping and changing tubing, similar to corticosteroid injections. All procedures were performed in normal clinical lighting conditions and ambient temperature. Briefly, this method consisted of a collection of 45 mL of blood, processed to result in 9–15 mL of neutrophil‐poor PRP. This injectate volume is larger than that most commonly reported by sports medicine physicians for knee injections (5–7 mL) [14], but much less than the osteoarthritic knee can hold (greater than 50 mL) [19]. The whole blood was collected into three 20 mL syringes containing ACD‐A anticoagulant and were capped. No platelet activators were used. The flange and the plunger of the syringes were clipped off with shears and placed into a Cole‐Parmer (model 17414‐21) centrifuge with counterbalance. The centrifuge was run at 750G for 5 min. The three ‘centrifuged’ syringes were then removed; a ‘PRP’ syringe was attached to a three‐way stopcock and the centrifuged syringes were consecutively added to the other end of the stopcock. The upper (plasma) portion of an upright centrifuged syringe was then shunted into the PRP syringe, which was repeated for all three syringes. No more than 5 min elapsed between stages of the procedure. No more than 30 min elapsed between completion of PRP preparation to injection; during that time, the PRP was placed on a rocker to avoid layer separation.

#### LC‐PRP injections

Injections were performed at day 0 and 3 weeks, for a total of two injections. This involved an ultrasound‐guided intra‐articular knee injection by an experienced sports medicine provider (at least 5 years postfellowship) after local anaesthesia with 1% lidocaine to the extra‐articular structures, namely the skin and joint capsule, to increase comfort. Intra‐articular injection of anaesthetic was avoided. Arthrocentesis was performed before injection if an effusion was present.

#### Blood analysis

All subjects had less than 1 mL of their whole blood and PRP analysed, with a complete blood count (CBC) with differential separately on each.

All subjects were instructed to avoid nonsteroidal anti‐inflammatory medications for 1 week before and 2 weeks after the injection to avoid impaired platelet function and subsequent diminished therapeutic benefit from the PRP injection [26]. Subjects were recommended to avoid strenuous activity for a day but were allowed to walk as normal following the procedure. Additionally, subjects were recommended to further avoid strenuous activity if they felt fullness or pain following the procedure, until it returned to a tolerable level.

### Measures

#### LC‐PRP composition

The PRP cellular composition of interest was platelet extraction/yield (defined as the PRP platelet volume divided by the whole blood platelet volume)—a higher platelet extraction means a higher number of total platelets are preserved. Prior literature has demonstrated commercial neutrophil‐poor PRP kits to produce platelet extraction (platelets in PRP divided by platelets in the whole blood) of around 0.30–0.60 (mean 0.45) [32]. Platelet count, concentration and concentration factor were recorded. Additionally, white blood cell (WBC), red blood cell (RBC) and haemoglobin (Hb) concentrations were also recorded.

#### Patient‐reported outcome measures

In accordance with the Osteoarthritis Research Society International (OARSI) guidelines [34], the following information was collected via REDCap [23]; age, gender, body mass index (BMI), duration of symptoms, prior knee surgeries/injections, analgesic medication usage in the last 3 months, comorbidities, affected knee compartment(s), K–L score, baseline Western Ontario and McMaster Universities Osteoarthritis Index (WOMAC) score (total, functional and pain measure), patient global assessment score and global patient satisfaction (GPS). Follow‐up data were collected at 1, 3, 6 and 12 months after the second injection.

#### Patient safety and adverse events

Patients were instructed to call if they experienced any adverse events. Safety was also tracked via a safety review performed at the primary endpoint of 6 months.

### Economic evaluation

#### Cost

Cost per injection was calculated as follows. For PRP, the total cost per injection consisted of: (1) the cost of medical assistant time required to prepare the LC‐PRP (on average over the course of the study) in addition to (2) the cost of LC‐PRP preparation supplies. These included: tourniquet ($0.25), butterfly venipuncture kit ($0.50), three‐way stopcock ($0.50), syringe cap ($0.25 × 3), syringes ($0.25 × 4), anticoagulant (ACD‐A, $1.00) and medical assistant time ($21/h). Corticosteroid injection was calculated simply as the cost of the vial of triamcinolone acetonide, 1 mL of 40 mg/mL, estimated at $10. Thus, costs were similar for the injectate, but did not take into account facility fees, physician fees, standard injection supplies and so on. As previously mentioned, primary clinical endpoints were 6 months (two LC‐PRP injections, or two cortisone injections).

#### Cost‐effectiveness modelling

For a more in‐depth economic evaluation, we constructed a Markov simulation model to conduct a cost‐effectiveness analysis (CEA) of LC‐PRP compared to corticosteroid injection. This model used the third‐party payer perspective and had a time horizon of 12 months, based on two cortisone injections or two LC‐PRP injections. Cost inputs for this simulation model consisted of intervention costs as well as the reimbursement cost to the payer for the procedure (see Supporting Information S1: Appendix 2). The current total cost for a corticosteroid injection includes: medication, physician fee, facility fee and supplies (needles, syringes, etc.). We assumed that patients in this arm would receive a corticosteroid injection every 3 months (standard of care). The costs for LC‐PRP include physician fee, facility fee and supply cost (see Supporting Information S1: Appendix 2). Unlike corticosteroids, however, there is no medication cost. An additional cost of LC‐PRP is the time for medical assistants to perform the procedure. We monitored and recorded the amount of time it takes for each task (venipuncture, centrifuge process time and procedural time) and then multiplied this by the hourly wage rate of a medical assistant to get an estimate of the value of this time. Physician and facility fees were obtained from Medicare. EuroQOL (EQ‐5D) questionnaires were also administered, which were used for constructing quality‐adjusted of life‐years (QALYs), a commonly used effectiveness measure in economic evaluations which incorporate both quality and quantity of life [3, 4]. We used this model to estimate the expected cost and expected effectiveness for both strategies and then constructed an incremental cost‐effectiveness ratio (ICER) using a willingness‐to‐pay (WTP) threshold of $100,000/QALY. We then performed probabilistic sensitivity analyses to explore the robustness of our assumptions using 10,000 Monte Carlo simulations in which each parameter value was drawn from a distribution. We used gamma distributions for cost parameters and beta distributions for utility parameters.

### Statistical analysis

Whole blood and LC‐PRP outcomes (e.g., platelet concentration, platelet count) were summarised with descriptive statistics including mean and standard deviation. Our calculated LC‐PRP platelet extraction rate was compared to platelet extraction rates from other PRP preparation techniques reported in the literature via 95% confidence intervals [32]. Differences in effectiveness outcome measures (e.g., total WOMAC, pain WOMAC, functional WOMAC, global assessment score) between baseline and follow‐up (e.g., 1 month, 3 months, 6 months, 12 months) were evaluated using Wilcoxon signed‐rank tests. Tests were two‐sided, and a significance level of 0.05 was used. Statistical analyses were performed using SAS version 9.4 (SAS Institute, Inc.).

## RESULTS

**Figure 2:**
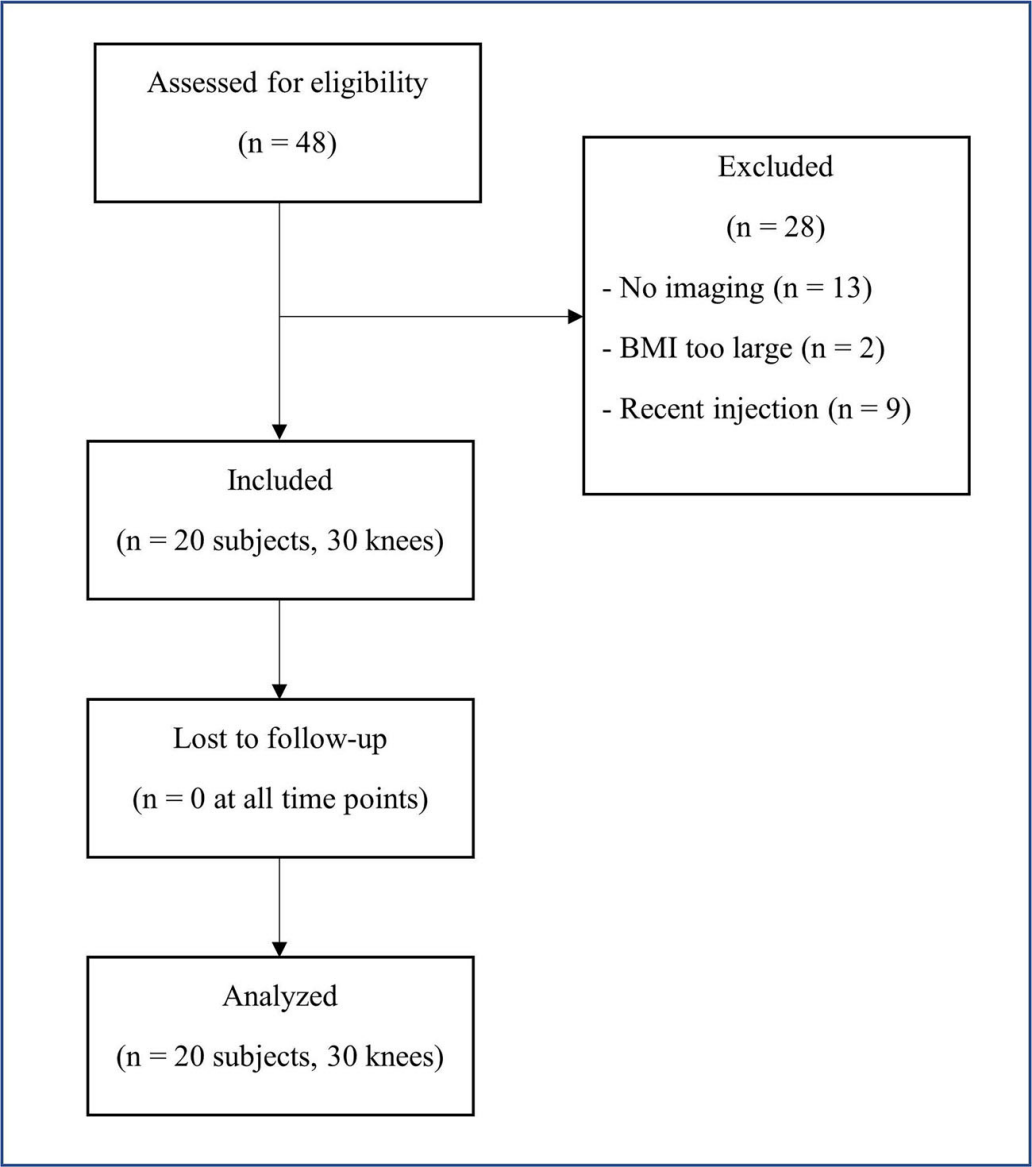
Flowsheet of subjects approached for study. BMI, body mass index; K–L, Kellgren–Lawrence grade of osteoarthritis.

Frequencies and percentages of patient characteristics are presented in Table 1. We enrolled a total of 20 patients (30 knees). The mean (standard deviation) duration of symptoms was 11.9 (12.3) years. Eleven knees were Kellgren–Lawrence grade 1 (37%), 12 were grade 2 (40%) and 7 were grade 3 (23%), representing a spectrum of mild to moderate knee OA. Figure 2 demonstrates the subject flowsheet for the study, illustrating that no patients were lost to follow‐up, as 100% of data were received at all follow‐up time points. One patient (two knees) elected to have only one injection and was included in the analysis.

**Table 1:** Demographic information for all subjects enrolled in study.

		*n*	%	Mean	SD
*n*, patients		20			
*n*, knees		30			
Age				48.8	14.2
Body mass index (kg/m^2^)				26.5	4.1
Duration of symptoms (years)				11.9	12.3
Female sex		12	60		
Knees injected					
Bilateral		10	50		
Left		14	47		
Right		16	53		
Remote prior corticosteroid injection		6	30		
Prior knee surgery		12	60		
Opioids in last month		1	5		
Kellgren–Lawrence grade					
1		11	37		
2		12	40		
3		7	23		

### PRP composition

**Figure 3:**
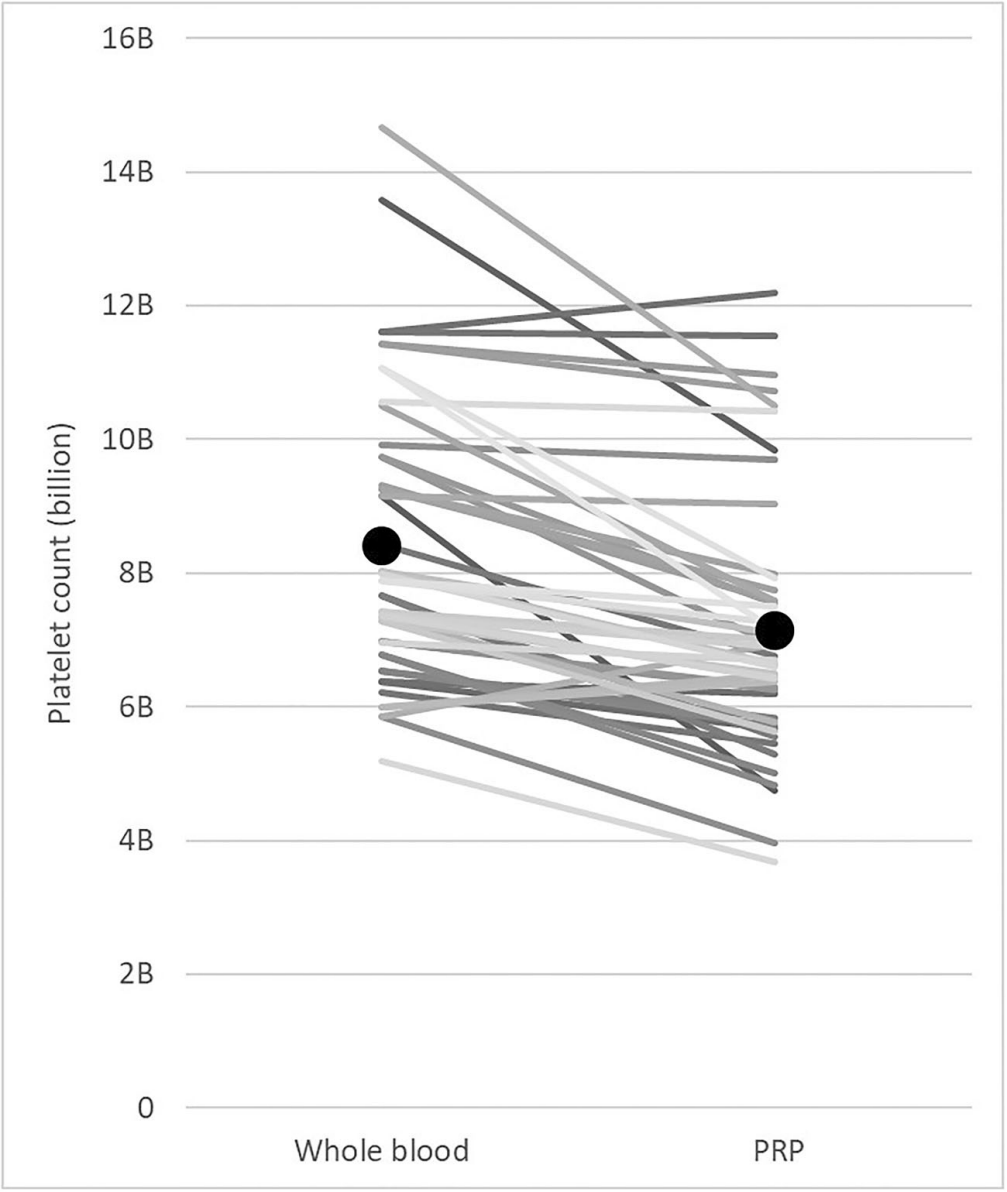
Platelet extraction rates/yields are demonstrated for all subjects (individual lines). Average values for all subjects are noted as black circles.

**Figure 4:**
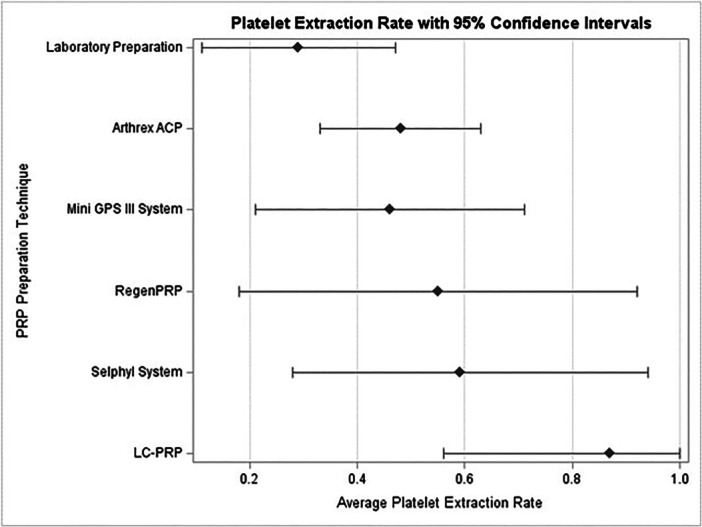
Average platelet extraction rate, and corresponding 95% confidence intervals, by PRP preparation technique. Note: Estimated extraction rates and 95% confidence intervals for all methods but LC‐PRP were calculated by Magalon et al. [32]. LC‐PRP, low‐cost platelet‐rich plasma.

Whole blood and PRP cellular analysis were performed on all subjects at each of the two injections. Three subjects (three knees) had inaccurate whole blood values recorded on one of their injections, related to operator error; these values were discarded and not included in the analysis. All PRP cellular analysis values were accurately recorded. Table 2 displays the blood parameters for whole blood and PRP while Figure 3 demonstrates platelet extraction rates for all injections. On average, approximately 8.4 billion platelets were present in the whole blood, and 7.1 billion platelets were obtained within the PRP (85.0% extraction rate). Figure 4 demonstrates platelet extraction rates for LC‐PRP compared to other PRP preparation techniques (e.g., commercial kits) as documented in the literature [32]. Mean injected PRP volume was 14.9 mL (SD 3.0). White blood cell concentration in the PRP averaged 3.3 (SD 1.9) x10^3^ per μL, haemoglobin concentration of 0.1 (0.0) g/dL, and a platelet concentration of 485.4 (SD 117.6) x 10^3^ per μL.

**Table 2:** Whole blood and platelet‐rich plasma parameters (*n* = 20 patients, 30 knees).

	Whole blood		Platelet‐rich plasma	
	Mean	SD	Mean	SD
Volume	45.0	0.0	14.9	3.0
Platelet concentration, 10^3^ per μL	183.0	51.4	485.4	117.6
Platelet count (billion)	8.38	2.07	7.12	2.06
Platelet extraction (%)			85.0%	
Concentration factor			2.7 x	
White blood cell concentration, 10^3^ per μL	4.9	1.2	3.3	1.7
‐ Neutrophils	3.1	1.0	0.08	0.1
‐ Lymphocytes	1.4	0.3	2.6	1.4
Red blood cell concentration, 10^6^ per μL	4.0	0.5	0.0	0.0
Haemoglobin concentration, g/dL	13.0	1.5	0.1	0.0

### Patient‐reported outcomes

**Figure 5:**
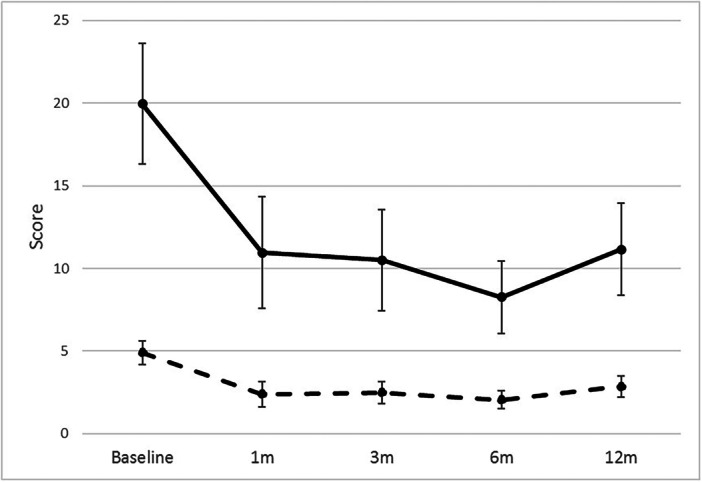
Western Ontario and McMaster Universities Osteoarthritis Index (WOMAC) score over the study. The solid line denotes the total WOMAC score, while the dashed line indicates the WOMAC pain subscore.

Figure 5 displays the total WOMAC score and WOMAC pain score over the study period for all subjects. Significant differences in WOMAC score (1 month: *p* < 0.0001; 3 months: *p* < 0.0001; 6 months: *p* < 0.0001; 12 months: *p* = 0.0149) and WOMAC pain subscore (1 month: *p* < 0.0001; 3 months: *p* = 0.0003; 6 months: *p* = 0.0001; 12 months: *p* = 0.0030) were noted (Table 3). Significant differences in WOMAC functionality subscore (1 month: *p* = 0.0001; 3 months: *p* < 0.0001; 6 months *p* < 0.0001; 12 months: *p* = 0.0410) and global assessment score (1 month: *p* = 0.0010; 3 months: *p* < 0.0001; 6 months *p* = 0.0015; 12 months: *p* = 0.0013) were also observed at all follow‐up points compared to baseline (Table 3). Prior to the injections, 85% of patients noted that they were unsatisfied with their knee(s), which became 40% at 1 month, 35% at 3 months, 15% at 6 months and 25% at 12 months. Satisfaction with the procedure was noted in 75% of patients at 1 month, 80% at 3 months, 90% at 6 months and 75% at 12 months. No serious adverse events were reported—20% noted an increase in knee fullness/swelling and 15% noted pain in addition to fullness/swelling, all of which lasted 48 h.

**Table 3:** Western Ontario and McMaster Universities Osteoarthritis Index (WOMAC) score and WOMAC subscores at baseline and follow‐up (i.e., 1 month, 3 months, 6 months, 12 months).

	Baseline	One month	*p*‐value^a^	Three months	*p*‐value^a^	Six months	*p*‐value^a^	Twelve months	*p*‐value^a^
	Median (interquartile range)	Median (IQR)		Median (IQR)		Median (IQR)		Median (IQR)	
WOMAC total score	19.5 (12.5)	7.0 (7.0)	<0.0001	7.0 (6.5)	<0.0001	5.5 (9.5)	<0.0001	6.5 (11.5)	0.0149
WOMAC pain subscore	4.0 (3.0)	1.0 (2.0)	<0.0001	1.5 (2.0)	0.0003	1.5 (3.0)	0.0001	1.5 (3.0)	0.0030
WOMAC functionality subscore	11.5 (9.0)	4.0 (6.5)	0.0001	4.0 (4.0)	<0.0001	4.0 (6.5)	<0.0001	3.0 (8.5)	0.0410
Global assessment score	60.0 (15.0)	70.0 (15.0)	0.0010	75.0 (10.0)	<0.0001	80.0 (25.0)	0.0015	70.0 (25.0)	0.0013

^a^ Wilcoxon signed‐rank test.

### Feasibility

Regarding feasibility, key times were recorded for when the blood draw process began and when LC‐PRP processing was complete for all 58 injections. Using these time measures, we found that the average time required for processing LC‐PRP in this study was 20 min. No patients required repeat blood draws for this study.

### Cost and CEA

As previously noted, the injectate price per injection of corticosteroid was $10 compared to the price per injection of LC‐PRP of $11. From our more rigorous CEA, we observed, in our base case analyses, the LC‐PRP strategy (two injections) was both less costly ($654 vs. $1308) and more effective (0.846 QALYs vs. 0.708 QALYs) compared to the corticosteroid injection strategy (two injections for payer comparability), meaning that LC‐PRP was the dominant strategy. Results from the probabilistic sensitivity analysis are presented as a scatterplot on the cost‐effectiveness plane in Supporting Information S1: Appendix 3. The LC‐PRP strategy was the dominant strategy in each of the 10,000 iterations of the probabilistic sensitivity analyses.

## DISCUSSION

This pilot study demonstrates that a safe, low‐cost method of preparing PRP allows for recovery of 85% of platelets. Clinical outcomes at the primary endpoint, 6 months after the second of two injections, demonstrated significant clinical and statistical improvement for pain and function in this cohort of subjects with mild to moderate knee OA. No adverse events or serious adverse events were reported, and 90% of subjects reported satisfaction with the injection at the primary endpoint of 6 months. The cost of injection was significantly lower than previously published PRP values, and LC‐PRP was both less costly and resulted in more QALYs than corticosteroid injection. Finally, this process was shown to be feasible in clinical settings, as evidenced by the lack of repeated blood draws required and the average 20‐min LC‐PRP processing.

### Comparison with prior literature

Prior studies have examined lower‐cost methods of PRP [1, 10, 17, 27, 31, 44], but most studies conducted have been retrospective, lacking full cellular analysis, or have not been conducted in populations of patients with knee OA. Additionally, like the present study, the sample sizes associated with these investigations are limited, and the protocol is nonrandomized. Although PRP preparation varies between these lower‐cost method PRP studies, many researchers observed similar concentration factors in their PRP yield as we found through our study, ranging from 1.17 to 3.09 [1, 17, 31]. We were unable to compare platelet recovery with these studies. Fernandez‐Fuertes et al., who conducted the only other study on lower‐cost PRP preparation for knee OA to date observed that WOMAC scores improved, and basic satisfaction was achieved in 70% of patients [17]. Thus, although the literature on LC‐PRP, especially in the context of knee OA, is scarce, these injections have been documented in the literature to be effective and well tolerated with no adverse events [17].

### Composition characteristics of LC PRP

The preparation technique described in this paper requires a centrifuge and basic medical equipment. Much of the prior PRP literature revolves around platelet concentrations, suggesting that higher concentrations are superior [16, 36]. However, total platelet count (dose) in the injectate appears to be a better marker of PRP quality as concentrations are merely a fraction of platelets per volume [7]. For example, 10 billion platelets in 10 mL is the same concentration as 10 platelets in 10 picoliters; one can imagine that an injection of 10 billion platelets would be more likely to provide clinical benefit. This study utilised a large volume, allowing for the administration of a higher number (dose) of platelets. An additional benefit of larger volume injections with more plasma is the higher yield of growth factors that reside in the plasma.

The total extraction rate (number of platelets in the PRP divided by the number of platelets in the whole blood) was around 85%. This is superior to the 45‐60% reported in commercial kits [32]. Furthermore, the platelet counts of 7.12 billion far exceeded the 1.25–3.61 billion seen by the same kits [32], and exceeds mean platelet dose of 5.5 billion used in RCTs demonstrating positive outcomes in knee OA treated with PRP injection [7]. This difference is likely accounted for by three factors: a higher volume of PRP, the inclusion of a small number of WBCs, and precise control over the removal of the plasma. Knees can withstand larger amounts of fluid than small joints, thus this technique may not be applicable to all joints or musculoskeletal pathologies. However, this modified syringe method can be performed as a double‐spin technique to formulate a PRP with a higher platelet concentration at minimally increased cost for smaller joints.

The composition of the PRP appeared to be beneficial as well. The presence of RBCs within joint injectates is universally seen as deleterious, as noted by iron‐induced cartilage and synovium injury [43]. The LC‐PRP in this study contained very few RBCs and a low Hb value, far lower than commercial kits [32]. The level of WBCs was minimally reduced by this technique. A recent meta‐analysis comparing leucocyte‐rich and leucocyte‐poor PRP for knee OA showed no difference between groups [38]; thus, this technique erred on the side of including more WBCs in order to obtain more platelets. The neutrophil count was significantly lower than that seen in commercial kits [18], which may be beneficial as neutrophils generate reactive oxygen species and metalloproteinases which may not be beneficial to the healing process [29]. The PRP low neutrophil count may have also contributed to the absence of postinjection complications such as pain/synovitis. One definition of leucocyte‐rich is when the PRP WBC concentration is higher than that of the whole blood [30]; thus, the LC‐PRP in this study can still fit the definition of leucocyte‐poor.

### Clinical outcomes

Clinical outcomes in this study were positive for both pain and function. A clinically significant WOMAC change at 6 months is 6.6 [52]; the patients in this study demonstrated improvements far above that level, even 1 year out. Many systematic reviews have demonstrated the efficacy of PRP in knee OA [5, 15, 38, 39, 50], though this is typically performed without a cost‐effective technique. Importantly, the large dose of platelets included in this study are in line with more recent studies, which have shown that larger amounts of platelets tend to give better clinical results [7, 40, 50]. As this study was not a RCT, a causal relationship between LC‐PRP injection and the observed improvements has not been proven, but the improvement in WOMAC is consistent with that seen in meta‐analyses of RCTs [54]. It is well known that intra‐articular injections of inert substances (e.g., saline) can produce significant beneficial effects in the knee [45], thus the true beneficial effect of the LC‐PRP compared to the act of the injection would need a RCT design to fully assess.

### Cost and cost‐effectiveness

As the cost of LC‐PRP was nearly identical to a vial of corticosteroid, this ideally will open doors for patients for a low‐cost treatment method without the known adverse effects of cortisone. Payors may recognise the cost savings, coupled with the copious clinical evidence of PRP's superiority to corticosteroids [24, 38, 47] to cover this treatment option.

Several previous studies investigating the cost‐effectiveness of PRP for knee OA have been published recently. One such study compared PRP to hyaluronic acid (HA) injections and found that PRP was associated with both higher costs and higher QALYs with an incremental cost‐effectiveness ratio of $12,000/QALY [46]. An additional study compared PRP to total knee arthroplasty using a societal perspective over a lifetime time horizon [42]. They found that PRP was not a cost‐effective strategy but cited a ‘lack of established clinical efficacy [for PRP] in relieving pain and improving function’ as a reason for this finding. However, this study did not identify cost‐effectiveness for PRP because the authors used standard PRP ($728) as the cost, which has an obvious detrimental effect on cost‐effectiveness. Finally, in a related study which undertook a systematic review of RCTs, authors identified a price of $1192 for PRP to be cost‐effective at a WTP threshold of $50,000/QALY compared to HA or saline injectable [6]. Our study contributes to the literature by incorporating prospectively collected utility scores derived from EQ‐5D into an economic evaluation of PRP and identifies dominance in cost‐effectiveness compared to standard‐of‐care.

### Limitations and future research

This study has limitations. Primarily, as this study was a prospective cohort study and not a RCT, our study is unable to draw causal conclusions about the LC‐PRP treatment; lack of blinding for patients or evaluators may introduce bias. More rigorous research is needed to evaluate causal relationships associated with LC‐PRP injection. Relatedly, the limitations of this study include a relatively small sample size. Additionally, we were not able to compare this preparation method with an already validated kit as part of our study design, or conduct a growth factor/cytokine analysis. The generalisability of our findings is limited as we had a homogenous sample, all recruited through a single health system. Our study included patients with mild to moderate (K–L 1–3) knee OA. The generalisability of the findings to patients with severe knee OA (K–L 4) is unknown. An additional limitation of our study is the loss of data due to operator error, as inaccurate whole blood values were recorded for three subjects due to clotting of the CBC samples. Finally, some platelet extraction rates were greater than one, suggesting inaccuracy in a few of the samples; this is likely related to machine noise in these patients. Previous experimental data performed on previous samples from other patients showed similar extraction rates when measured by a different cell counter.

As this pilot study establishes the feasibility of this LC‐PRP technique within a sample of patients with knee OA, RCTs can be performed to assess the efficacy of LC‐PRP for knee OA. Future studies could also evaluate the feasibility and efficacy of LC‐PRP within different patient populations (i.e., patients with other musculoskeletal disorders, patients with severe knee OA or with OA in different joints). Further, this study could be replicated at other sites, with larger sample sizes and more diverse samples.

## CONCLUSION

In conclusion, this pilot study demonstrates that LC‐PRP ($11) may benefit patients with knee OA. Our preliminary results show that pain and functionality improved, and patients tolerated the procedure with no serious adverse events reported. In total, 90% of patients reported satisfaction with the injection. Additionally, we found that this low‐cost method of preparing PRP allowed for recovery of 85% of platelets, which exceeded that of commercial kits. Overall, this new approach is cost‐effective compared to the standard corticosteroid injection with an ICER well below the WTP threshold of $100,000/QALY. The results of this study justify further investigation of this treatment within the population of patients with knee OA and can guide the design of future prospective RCTs.

## AUTHOR CONTRIBUTIONS

**Daniel M. Cushman**: Study design; data collection; data analysis; manuscript preparation. **Luke A. Johnson**: Data collection; manuscript review. **Taylor Burnham**: Study design; manuscript review. **Richard Nelson**: Study design; data analysis; manuscript preparation. **Jamie Egbert**: Data analysis; manuscript preparation. **Robert Burnham**: Study design; manuscript review.

## CONFLICT OF INTEREST STATEMENT

Taylor Burnham, DO, is a consultant for Avanos Medical and has received grant funding from DIROS Technology (paid directly to the University of Utah). The remaining authors declare no conflict of interest.

## ETHICS STATEMENT

This study obtained institutional review board (IRB) approval (IRB_00173470) through the University of Utah IRB. Informed consent was obtained from all participants.

## Supporting information

APPENDIX 1 – LOW‐COST PLATELET‐RICH PLASMA (LC‐PRP) PREPARATION. Appendix 2: Inputs for simulation model used to conduct cost‐effectiveness analysis. Appendix 3. Scatterplot depicting results from probabilistic sensitivity analysis. *Note:* Each dot represents one of 10,000 Monte Carlo simulations depicting the difference in cost (incremental cost) and difference in QALYs (incremental effectiveness) between LC‐PRP and corticosteroid.

## Data Availability

The datasets generated during and/or analysed during the current study are available from the corresponding author on reasonable request.
