# Supplementary material for: An evaluation of a low‐cost platelet‐rich plasma for osteoarthritis of the knee: A pilot study
Source: J Exp Orthop. 2025 Sep 5;12(3):e70420. doi: 10.1002/jeo2.70420 (PMC12411927; doi:10.1002/jeo2.70420)
Supplement: Supplementary file 1 — APPENDIX 1 – LOW‐COST PLATELET‐RICH PLASMA (LC‐PRP) PREPARATION. Appendix 2: Inputs for simulation model used to conduct cost‐effectiveness analysis. Appendix 3. Scatterplot depicting results from probabilistic sensitivity analysis. Note: Each dot represents one of 10,000 Monte Carlo simulations depicting the difference in cost (incremental cost) and difference in QALYs (incremental effectiveness) between LC‐PRP and corticosteroid. [file JEO2-12-e70420-s001.pdf]

APPENDIX 1 – LOW-COST PLATELET-RICH PLASMA (LC-PRP) PREPARATION METHOD

Step	
1	Ensure name and date-of-birth of subject.
2	Put stickers on all syringes, ensuring proper name & DOB throughout procedure
3	Add 2mL of ACD-A into 3 x 20mL syringes

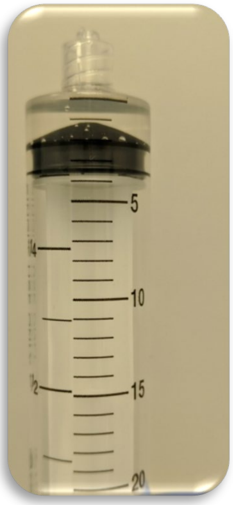

4	Venipuncture using butterfly needle

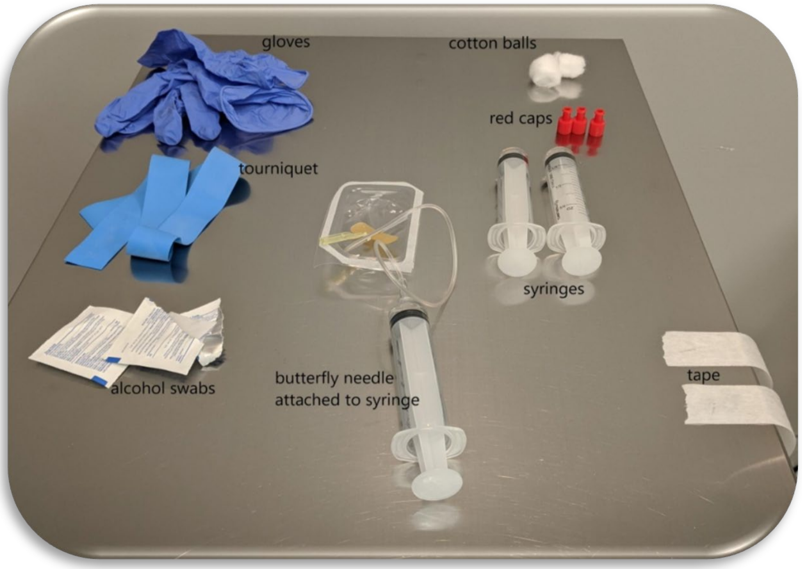

5 Draw 13mL blood (15mL total including 2mL ACD-A) into 20mL syringe - syringe 1

6 Draw 13mL blood (15mL total including 2mL ACD-A) into 20mL syringe - syringe 2

7 Draw 13mL blood (15mL total including 2mL ACD-A) into 20mL syringe - syringe 3

8 Draw 1-2mL blood into anticoagulated 3mL syringe for complete blood count (CBC) testing

9 If extra blood is drawn up on any needle, properly dispose of extra blood to leave exactly 15mL

10 Cap each syringe (qty 4) - 3 for PRP and 1 for CBC

11 Mix all syringes by themselves with a rocking motion, back and forth

12 Using shears, cut off the plunger and both flanges from each syringe

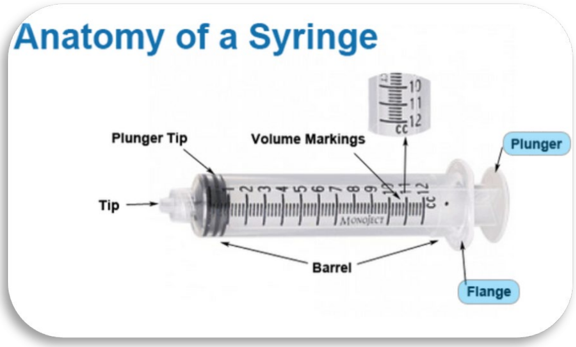

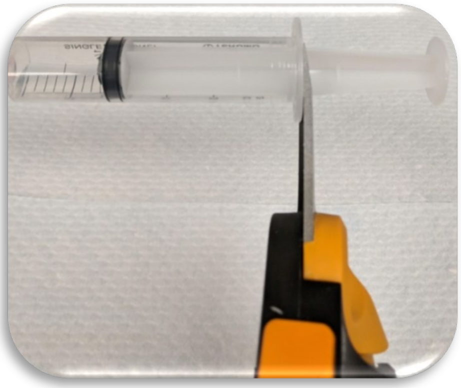

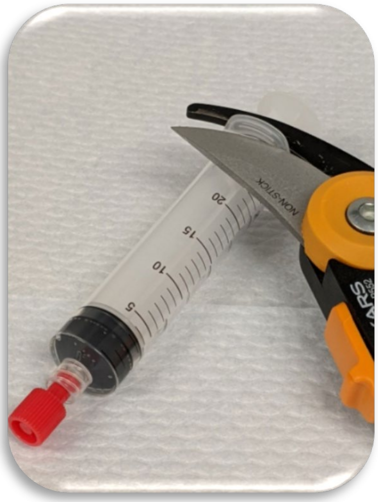

13 Place syringes in the centrifuge, opposite each other

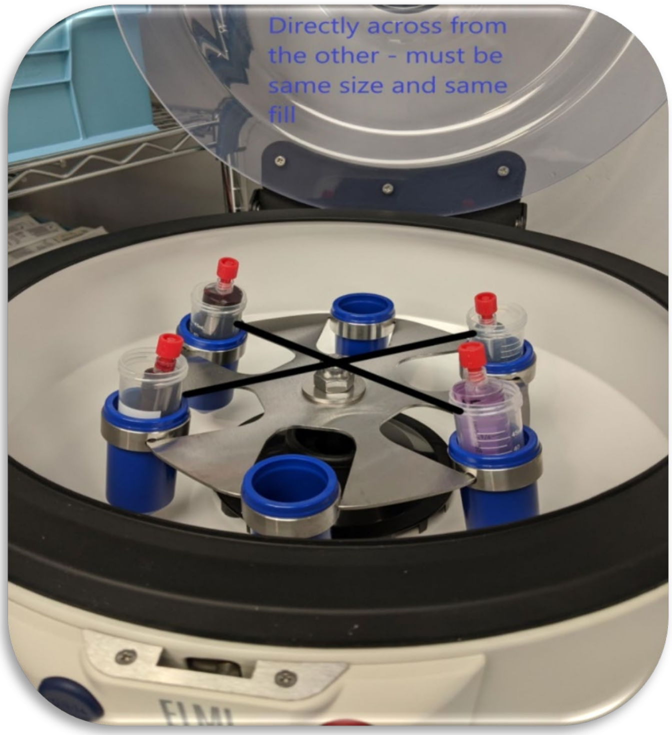

14 A fourth syringe will be used as a counterbalance, filled with 15mL of saline

15 Using a scale, syringes will be emptied (~0-0.5mL) to match weights for counterbalance

16 Close centrifuge lid

17 Centrifuge at 750G for five minutes

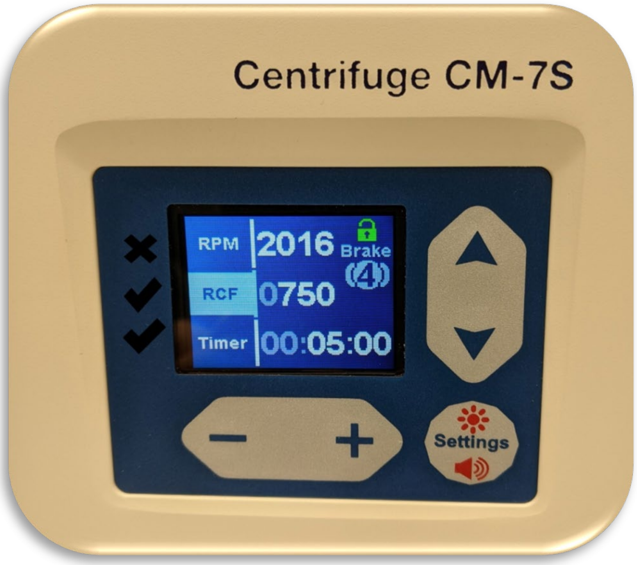

Ready stopcock - one "waste" syringe (20mL) and one "PRP" syringe (10mL)

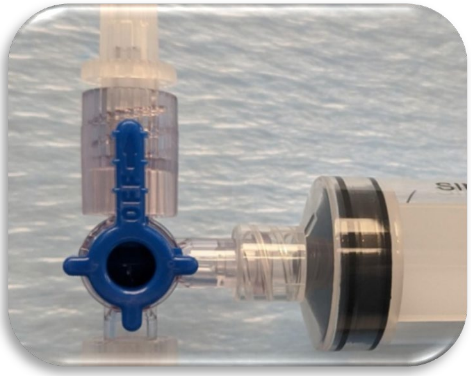

For each of 3 syringes, perform the following:

- Hold syringe upright and attach to bottom of stopcock

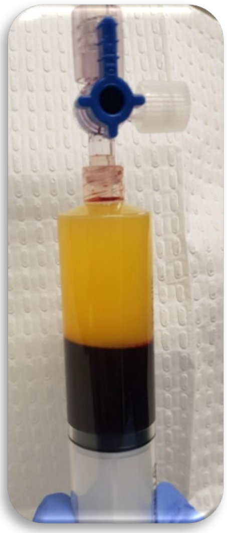

- (Optional, for lower volume, but lower platelet extraction) Distribute upper portion of plasma into "waste" syringe, leaving 3mL of plasma
- Rotate stopcock from "waste" syringe to "PRP" syringe
- Distribute remaining 3mL of plasma into "PRP" syringe

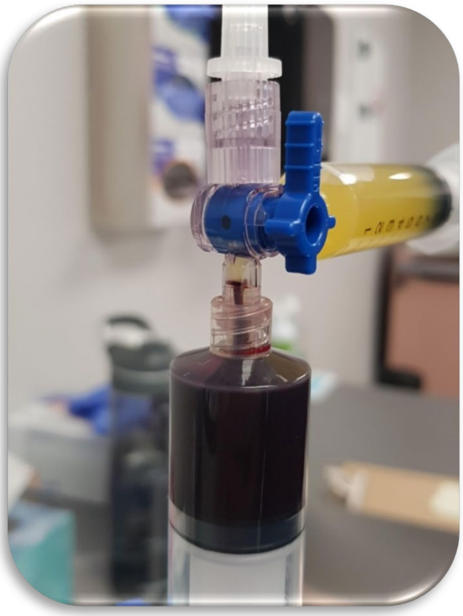

24 Repeat for each syringe. This will leave at least 9mL in the "PRP" syringe

25 Mix syringe with rocking motion (to ensure homogeneity)

26 From the "PRP" syringe, inject 1mL into separate 5mL syringe for CBC testing

27 This will leave at least 8mL in the "PRP" syringe

28 Physician will perform injection after local anesthetic administration, name/DOB verification, and sterile preparation

29 Perform CBC testing on both PRP and whole blood samples

Appendix 2: Inputs for simulation model used to conduct cost-effectiveness analysis

**Parameter**	**Corticosteroid value**	**PRP value**	**Source**
Costs			
Physician fee	$45	$45	Medicare
Facility fee	$271	$271	Medicare
Corticosteroid	$10	-	Assumption
Supplies	-	$5	Assumption
Medical assistant time (minutes)	-	17	Current study
Hourly wage for medical assistant	-	$21	US Bureau of Labor Statistics
Utilities			
Knee pain	0.69	0.69	Losina (2009), Brazier (2002), Losina (2013)
Corticosteroid injection	0.84		Losina (2009), Brazier (2002), Losina (2013)
LC-PRP			
Baseline	-	0.724	Current study
Month 1	-	0.775	Current study
Month 3	-	0.802	Current study
Month 6	-	0.792	Current study
Month 12	-	0.757	Current study

Note: The same CPT (20610) was used for both corticosteroid injection and LC-PRP

Appendix 3. Scatterplot depicting results from probabilistic sensitivity analysis

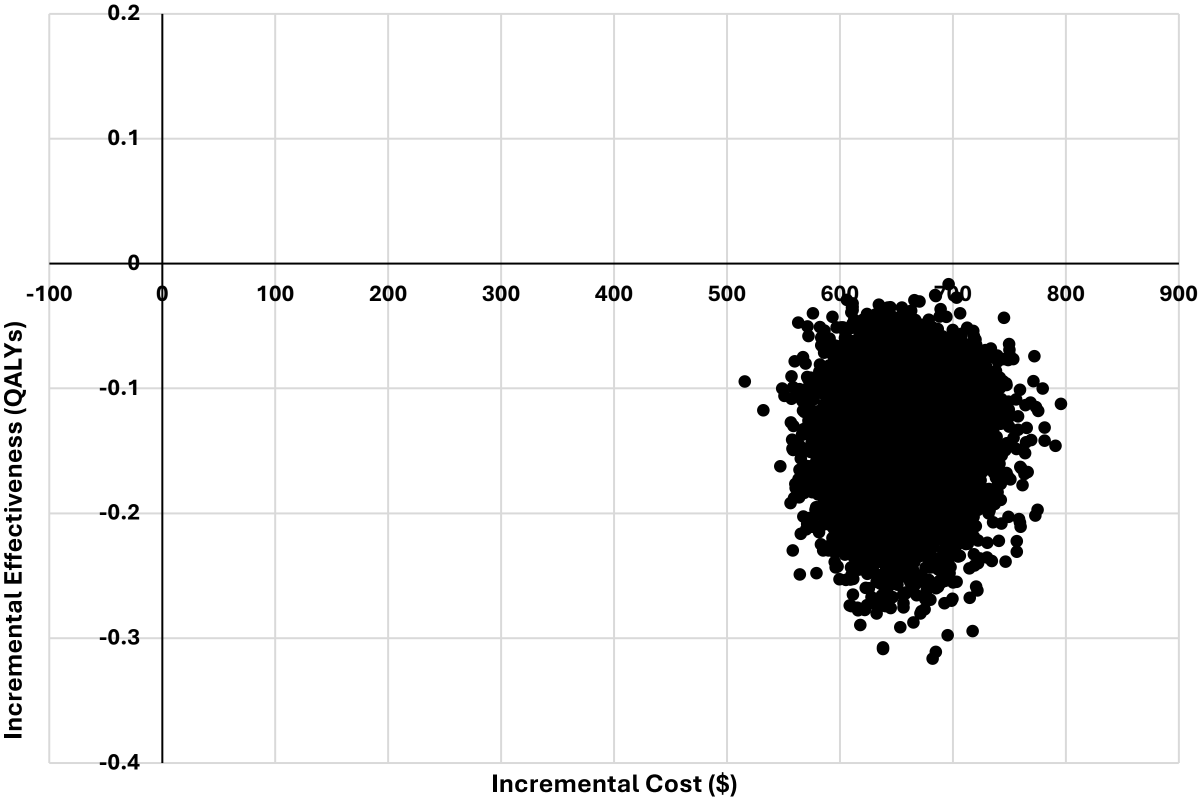

Note: Each dot represents one of 10,000 Monte Carlo simulations depicting the difference in cost (incremental cost) and difference in QALYs (incremental effectiveness) between LC-PRP and corticosteroid
